# Current status and development direction of immunomodulatory therapy for intervertebral disk degeneration

**DOI:** 10.3389/fmed.2023.1289642

**Published:** 2023-12-21

**Authors:** Yanbing Gao, Xiyue Chen, Guan Zheng, Maoqiang Lin, Haiyu Zhou, Xiaobo Zhang

**Affiliations:** ^1^Department of Orthopedics, Lanzhou University Second Hospital, Lanzhou, Gansu, China; ^2^Key Laboratory of Bone and Joint Disease Research of Gansu Province, Lanzhou, Gansu, China; ^3^Department of Orthopaedics, Sanya People’s Hospital, Sanya, Hainan, China; ^4^Department of Orthopedics, Honghui Hospital, Xi’an Jiaotong University, Xi’an, Shaanxi, China

**Keywords:** intervertebral disk degeneration, immunomodulation, immune cells, proinflammatory cytokines, intervertebral disk microenvironment, new options

## Abstract

Intervertebral disk (IVD) degeneration (IVDD) is a main factor in lower back pain, and immunomodulation plays a vital role in disease progression. The IVD is an immune privileged organ, and immunosuppressive molecules in tissues reduce immune cell (mainly monocytes/macrophages and mast cells) infiltration, and these cells can release proinflammatory cytokines and chemokines, disrupting the IVD microenvironment and leading to disease progression. Improving the inflammatory microenvironment in the IVD through immunomodulation during IVDD may be a promising therapeutic strategy. This article reviews the normal physiology of the IVD and its degenerative mechanisms, focusing on IVDD-related immunomodulation, including innate immune responses involving Toll-like receptors, NOD-like receptors and the complement system and adaptive immune responses that regulate cellular and humoral immunity, as well as IVDD-associated immunomodulatory therapies, which mainly include mesenchymal stem cell therapies, small molecule therapies, growth factor therapies, scaffolds, and gene therapy, to provide new strategies for the treatment of IVDD.

## 1 Introduction

**Figure 1:**
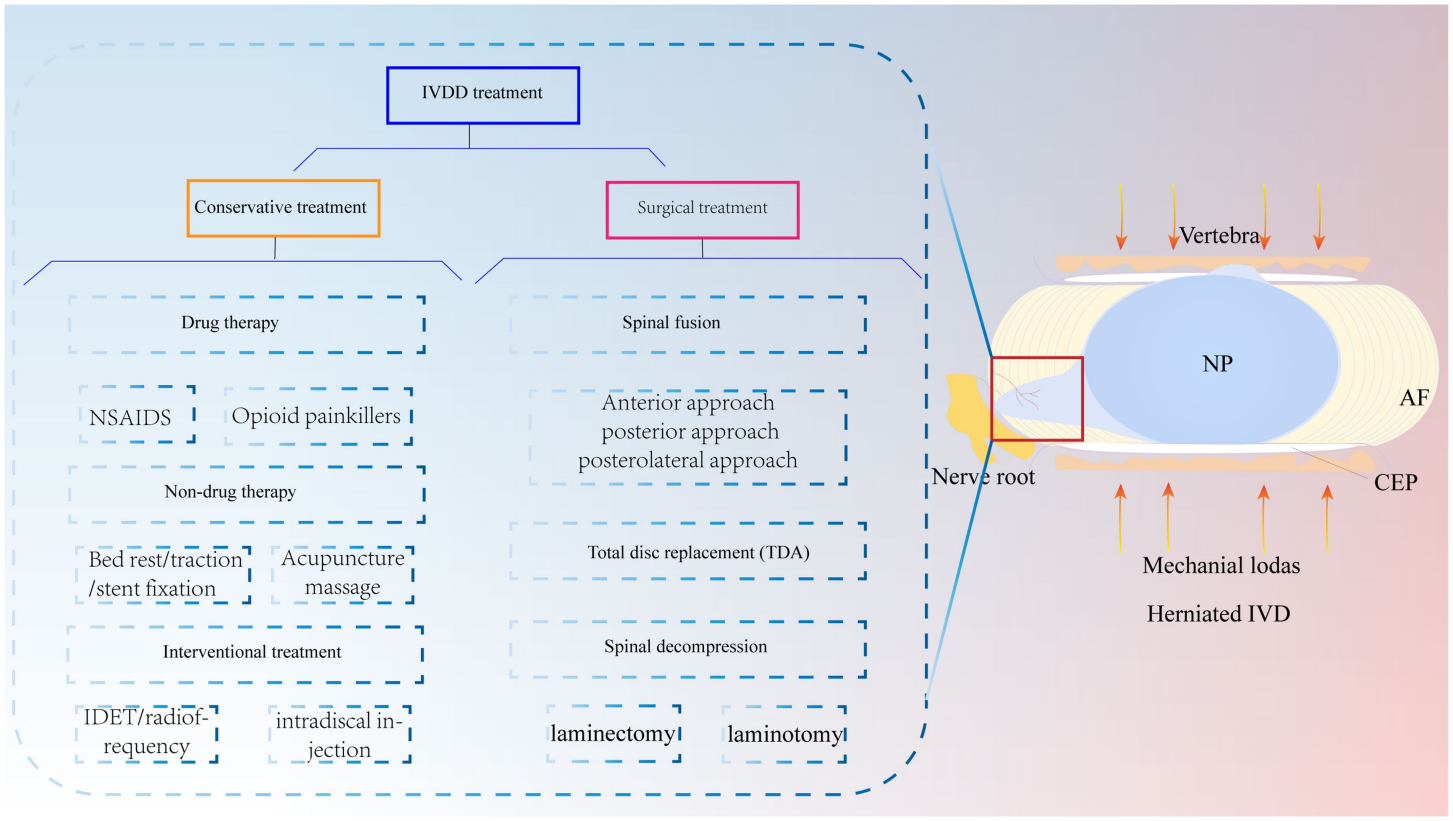
IVDD is currently treated clinically with both conservative and surgical treatments. Mechanical loading of the IVD is increased due to various reasons, which causes the NP to herniate and compress the nerve root to produce lower back pain. NP, nucleus pulposus; AF, annulus fibrosus; CEP, cartilaginous endplate.

In recent years, lower back pain has become an important social and medical problem worldwide and one of the causes of total disability in elderly individuals; this condition not only causes great pain and psychological burdens to patients and their families but also consumes a large amount of medical resources (1). Globally, it has been reported that approximately 60 to 80% of people will experience lower back pain symptoms (2). Among them, IVDD is regarded as one of the leading causes of lower back pain (3). Currently, conservative treatment of IVDD is preferred, including medication, bed rest, traction, stent fixation, acupuncture and massage, and interventional therapy (4, 5). Surgery is often chosen when patients exhibit acute neurologic deterioration or symptoms of cauda equina syndrome (6) (Figure 1). Of these, spinal fusion is the most common surgical intervention. However, surgery, which mainly relieves clinical symptoms, cannot cure the disease at the aetiologic level and severely destroys the stability, flexibility, and physiology of the normal IVD of the degenerative segmental spine (7). There are also complications, such as infections and nerve root compression (8). It has been shown that the loss of IVD tissue reduces IVD height and pressure and further accelerates the degeneration of adjacent phases of the spine (9). Thus, recurrent IVDD may also be a difficult problem for surgeons. Therefore, it is important to explore a novel and minimally invasive therapy to treat IVDD.

Recent studies on the relationship between immune regulation and IVDD have received much attention and focused on immune regulation and immune-related inflammation. It has been reported that IVDD-induced lower back pain is due to activation of the neuroimmune system, and activated immune cells release inflammatory cytokines, which send signals to the brain through neurohumours, causing pain (10). Some scholars have suggested that lower back pain is caused by radiculitis, which is triggered by the regulation of bioactive chemicals by the autoimmune response (11). Furthermore, inflammation and the immune response are inextricably linked and interact with each other. When IVD tissue is damaged and the exposed nucleus pulposus comes into contact with the immune system, an autoimmune response is triggered, causing macrophage infiltration and cytokine release (12). Additionally, monocyte chemoattractant protein 1 (MCP-1) released by the IVD promotes macrophage infiltration and amplifies the inflammatory response (13). Therefore, a full understanding of the immunomodulatory role of inflammatory cytokines can aid in the design of effective biologic therapies for IVDD. Therefore, this paper describes the structure and degenerative mechanisms of the IVD, examines the relevant role of modulating the immune response in IVDD, and proposes IVDD-related immunotherapy that combines mesenchymal stem cells, small molecules, growth factors, scaffolds, and genes with immunomodulatory roles to elucidate the role of the immune response and inflammation in IVDD. Understanding the mechanisms and therapeutic potential of immune inflammation in IVDD provides a theoretical basis for exploring new and more advanced strategies for treating IVDD.

## 2 IVD structure and mechanism of degeneration

### 2.1 IVD structure

**Figure 2:**
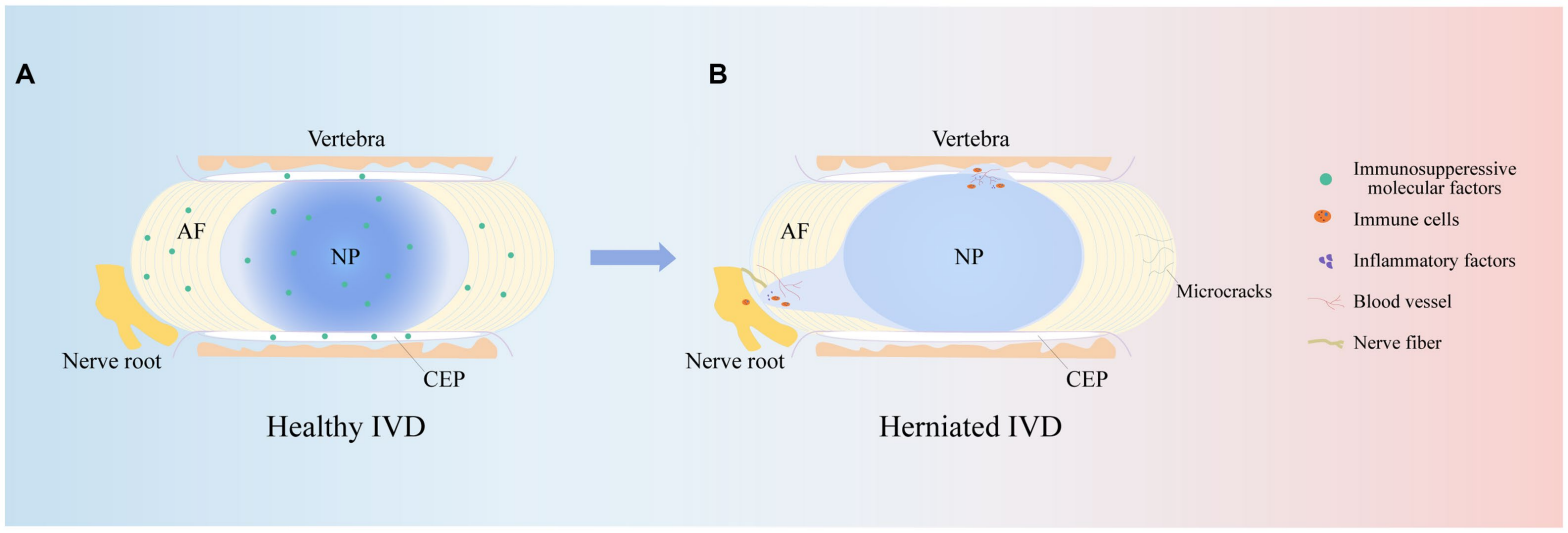
IVD structures. The herniated IVD compresses the nerve roots as well as the annulus fibrosus, and the microcracks in the upper and lower cartilage endplates stimulate an immune response, promoting immune cell and vascular infiltration, increasing nerve fiber innervation and secreting inflammatory factors, leading to clinical manifestations such as low backer pain and numbness.

The IVD is an avascular structure. It consists of the annulus fibrosus (AF), nucleus pulposus (NP) and cartilage endplates (CEPs). At the microscopic level, the IVD includes AF cells (AFCs), nucleus pulposus cells (NPCs), CEP cells and extracellular matrix (ECM), which are interdependent and collectively maintain the physiological functions of the IVD (14). CEP is a cartilaginous tissue containing proteoglycans and chondrocytes (15). It is the dividing line between the NP and the upper and lower vertebrae and a physical barrier against cellular and vascular infiltration (16). Therefore, maintaining CEP integrity is critical for protection against cytokine infiltration. The AF is the outermost fibrous cartilage structure of the IVD and is composed of collagen fibers, aggrecan and AFCs (17). The AF is divided into two layers: the outer layer is arranged at an angle of 65°, and the inner layer is arranged at 30°-45°. This structure can limit the expansion pressure and shear force of the NP (18, 19). The outer AF is crossed by sensory nerve fibers and blood vessels that help transport nutrients to AFCs in IVD tissue (20). The NP is mainly composed of type II collagen and aggrecan (21). Among them, aggrecan is negatively charged and forms a high osmotic pressure to maintain the gelatinous state of the NP (22). This plays an important role in maintaining IVD height. In summary, the complete IVD structure is responsible for spinal support, motion, weight bearing, durability, and flexibility (Figure 2A).

### 2.2 IVDD mechanism

It is well known that IVDD is an age-related degenerative disease, and its prevalence increases with age. Studies have shown that approximately 90% of patients over 50 years of age develop IVDD (23). This may be associated with cellular senescence, reduced cell proliferation, limited self-repair capacity and enhanced catabolism (24). In addition, the NP is supported by nutrients that diffuse from the upper and lower CEP to maintain cellular metabolism. Studies have shown that during IVDD, the IVD microenvironment becomes nutrient deficient, which inhibits NPC viability and proliferation and leads to NPC apoptosis (25). In addition to age and nutrient disorders, other risk factors for IVDD are smoking, obesity, unhygienic lifestyle, trauma and abnormal mechanical load (26). Abnormal mechanical loading, in particular, continuously squeezes the NP and causes it to herniate or bulge and affects macrophage function in the IVD microenvironment, leading to an excessive and uncontrolled inflammatory response (27, 28). Notably, varying degrees of inflammation result in the production of inflammatory cytokines, upregulate ECM-degrading enzymes, and initiate an immune response that stimulates an inflammatory response, which in turn exacerbates IVDD (29). Therefore, an adequate understanding of the risk and contributing factors for IVDD is essential for studying the pathogenesis of IVDD. Notably, the pathophysiology of IVDD is related to many variables, but its main feature is the disruption of anabolic and catabolic processes within the IVD. For example, there is a lack of anabolic factors, massive release of inflammatory cytokines and activation of ECM-degrading enzymes (30). This series of factors causes pathological changes such as oxidative stress, ECM catabolism, abnormal proliferation, autophagy, and the inflammatory response, resulting in a decrease in IVD cells, the loss of cell physiological function, and an imbalance in the synthesis and catabolism of the ECM, which in turn leads to a decrease in the water content of the NP, a decrease in AF hydration, and structural disorders of the AF, infiltration of the vasculature, an increase in innervation, the appearance of microcracks in the structure of the CEP, thinning, mineralization, and osteosclerosis, which ultimately leads to changes in the mechanical properties of the IVD, a decrease in the height of the IVD, and herniation of the NP (31–34). This ultimately causes compression of painful nerve fibers, leading to corresponding clinical symptoms (Figure 2B).

## 3 Immunomodulation associated with IVDD

### 3.1 Role of the immune response in IVDD

The immune response is the process by which the immune system recognizes and removes foreign substances. The human immune system consists of innate and adaptive immune responses (35). The innate immune response is an emergency response to noxious stimuli, which, when activated, is effective in avoiding tissue damage and excessive inflammatory responses (36). The adaptive immune response recognizes pathogens in a highly specific manner and is responsible for the formation of immune memory over time, and it typically results in lifelong immunity to reinfection by the same pathogen (37). In addition, innate and adaptive immunity do not operate independently, and adaptive immune responses occur in response to the innate immune response (32). Notably, chemokines, inflammatory cytokines and some immune cells constitute the first line of defense against harmful stimuli (38). As IVDD progresses, damaged IVD tissue will trigger an immune-inflammatory cascade, resulting in the secretion of a variety of inflammatory cytokines and chemokines and the recruitment of a variety of immune cells that become activated. Interestingly, these cytokines and immune cells produce proinflammatory mediators while clearing necrotic tissues (39), which in turn stimulate downstream inflammatory signaling pathways, leading to a disturbed IVD microenvironment and exacerbating IVDD.

#### 3.1.1 Inflammatory cytokines and chemokines in IVDD

Inflammatory cytokines and chemokines are proteins that have proinflammatory and renewal functions and coordinate the immune response to modulate the migration of immune cells (40). As the disease progresses, IVD cells abnormally secrete inflammatory cytokines and exhibit enhanced degradation of ACAN and COL-2, and the IVD cell phenotype changes (41). In addition, structural breakdown secondary to leakage of NP tissue through microscopic fissures in the AF recruit various immune cells to the damaged site to stimulate the immune response and continue to secrete inflammatory cytokines, leading to the onset of pain and exacerbating IVDD. Thus, inflammatory cytokines can alter the renewal and differentiation of IVD cells, which contributes to tissue repair and immune homeostasis. Chen et al. (42) studied NPC and found that an increase in the inflammatory cytokine IL-1β accelerated IVDD through NF-KB signaling and mitochondrial reactive oxygen pathways to form NLRP3 inflammatory vesicles, which are involved in the innate immune response. In contrast, IL-6 is an inflammatory cytokine that maintains homeostasis *in vivo*, is produced immediately in response to tissue injury in the organism and protects the host against this harmful stimuli through an immune response (43).

**Figure 3:**
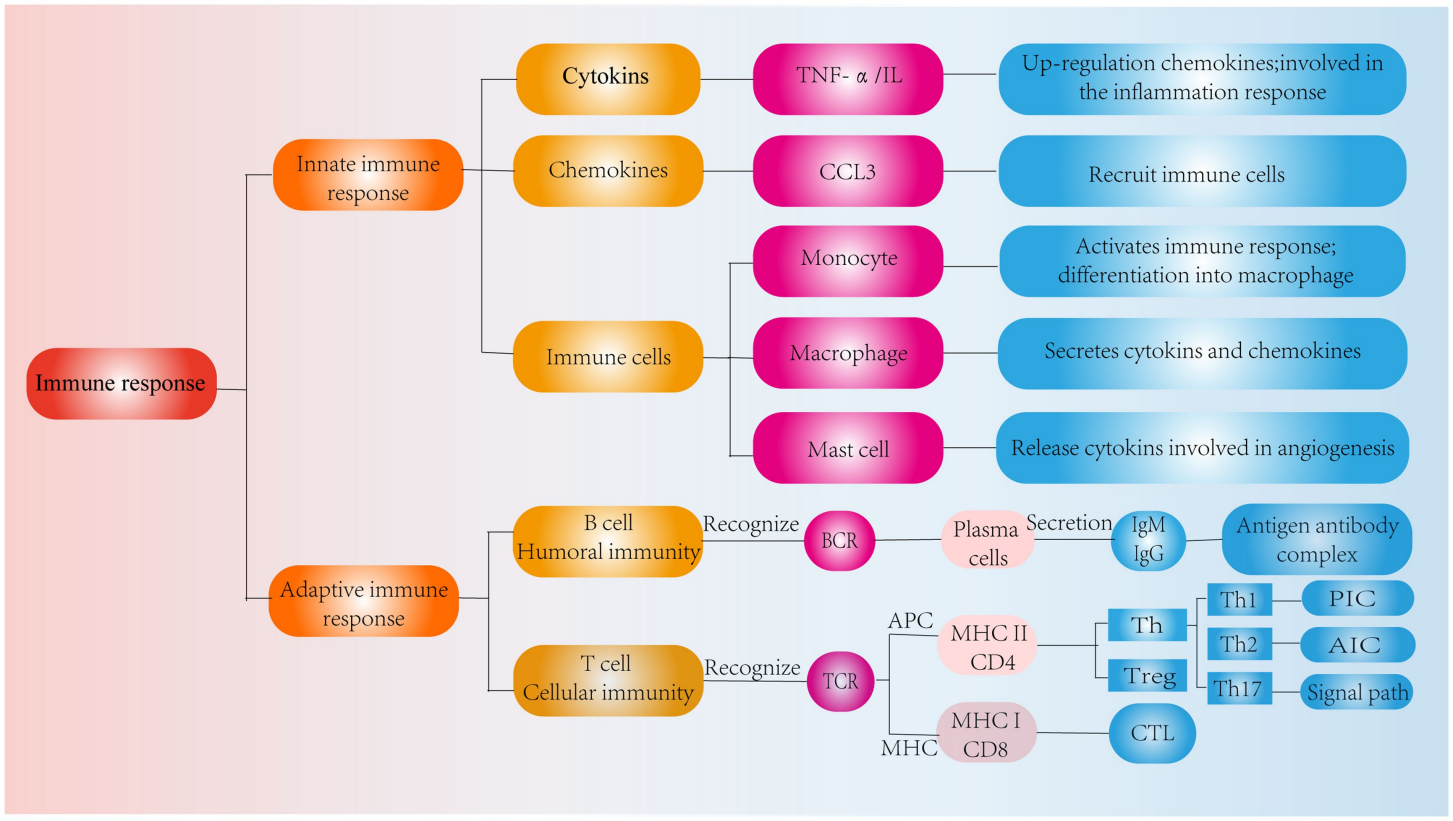
Classification of immune response. Role of cytokines, chemokines and immune cells in IVD tissues in the innate immune response and pathways of action of humoral and cellular immunity in the adaptive immune response.

In addition, chemokines are signaling proteins that can induce targeted chemotaxis in immune cells and coordinate interactions between immune cells (44). Some scholars have found that the induction of human NPCs with TNF-a upregulates the expression of CCL3, promotes macrophage infiltration into the IVD and aggravates IVDD (45). In parallel, the Th17 cell-derived inflammatory cytokine IL-17 also induces chemokine production, recruits monocytes to sites of inflammation, and amplifies the immune inflammatory response by acting synergistically with TNF-α and IL-1 (46). Thus, cytokines produced by degenerated IVD tissues can upregulate chemokine expression, recruit innate immune cells to sites of IVD damage, promote immune cell infiltration and activation, and directly or indirectly modulate the immune response during IVDD. These findings suggest that inflammatory cytokines and chemokines play important roles in recruiting immune cells into IVD-associated tissues and are important factors that contribute to IVDD and pain induction (Figure 3).

#### 3.1.2 Immune cell infiltration and activation in IVDD

In addition to degenerated IVD tissues, various immune cells migrate to the periphery of IVD tissues while producing inflammatory cytokines. These factors stimulate neovascularization through immune cell activation, nerve fiber growth and vascular infiltration into damaged IVD tissue, further amplifying the inflammatory response (26). In contrast, the affected immune cells are predominantly monocytes/macrophages, which have a wide range of immunomodulatory, inflammatory and tissue repair capabilities (47). Experiments have shown that the number of monocytes and macrophages is significantly increased in patients with IVDD (48). On the one hand, monocytes contain multiple receptors, such as Toll-like receptors and NOD-like receptors, which activate the immune response, release inflammatory cytokines and are directly involved in phagocytosis (49). On the other hand, during the inflammatory response, monocytes can differentiate into macrophages that stimulate and recruit additional immune cells to the site of inflammation (50), thus participating in the development of IVDD. In addition, mast cells play a nonnegligible role in inflammation. These cells release preformed granules containing cytokines during degranulation and are recruited into damaged IVD tissue to enhance angiogenesis (51). Wiet et al. (52) found that mast cells were specifically recruited to the degenerated IVD through the upregulation of stem cell factors. At this site, mast cells can induce NPC and CEP cell catabolism and proinflammatory phenotypes by interacting with IVD cells after degranulation, and many cytokines and vascular growth and neurogenic factors are released into the IVD microenvironment. It is evident that mast cell induction is involved in the immune inflammatory response and induces pain development. During IVDD, these immune cells are recruited to the site of IVD tissue inflammation, which promotes immune cell infiltration and activation and the secretion of many cytokines, and contribute to local vascular and nerve infiltration, leading to chronic inflammation and pain.

### 3.2 Innate immune response

#### 3.2.1 Toll-like receptors

Toll-like receptors (TLRs) activate multiple receptors in the innate immune response (53). They play crucial roles in the pathological mechanism of IVDD. It has been reported that TLRs can promote the secretion of inflammatory factors and protein hydrolases through related signaling pathways, leading to IVDD (54). TLR expression was upregulated in degenerated IVD tissues, especially TLR4. Recently, Wang et al. that Fer-1, which is an inhibitor of iron death, could downregulate TLR4 expression and thus inhibit the NF-KB pathway to alleviate IVDD in rats (55). In addition, Zheng et al. showed that the protein S100A8/A9 exacerbated IVDD by binding to TLR4 and activating the NF-KB signaling pathway to upregulate the expression of matrix metalloproteinases, TNF-α and IL-6 (56). Moreover, TLR4 is closely associated with the NLRP3 inflammasome. Studies have confirmed that activation of the TLR4/NLRP3 signaling pathway upregulates the expression of oxidative stress-related genes. Treatment with Ganoderic Acid A significantly upregulated ECM anabolic factors and inhibited the secretion of inflammatory factors and oxidative stress, thus protecting NPCs (57). Notably, Jacobsen et al. recently altered the biomechanical properties of NPCs by attenuating actin alterations with the TLR4 inhibitor TAK-242, thereby attenuating inflammatory injury (58). Thus, IVDD can be effectively alleviated by inhibiting the TLR4 inflammatory cascade, leading to signaling changes in cells. In addition to TLR4, TLR2 expression is progressively upregulated by IVDD and pain levels. In degenerated IVD tissues, the interaction of endogenous TLR2 ligands can activate the NF-kB signaling pathway and the MAPK pathway, which ultimately leads to lower back pain (59). In addition, TLR2 may be associated with IVD cell senescence. Studies have shown that the activity of TLR2 can be blocked by o-vanillin, which in turn reduces the number of senescent IVD cells and the secretion of senescence-related factors (60). In conclusion, inhibiting activation of the innate immune response and TLR is a potential strategy to slow IVDD and reduce chronic lower back pain.

#### 3.2.2 NOD-like receptors

When necrotic tissue is present or an inflammatory environment is present, a pattern recognition receptor (PRR) activates host defense against this noxious stimulus (49). The PRR recognizes the presence of invading microorganisms or noxious stimuli called damage-associated molecular patterns, which can trigger downstream inflammatory pathways to eliminate microbial infections and other noxious stimuli (61). Activation of inflammatory vesicles is a major factor in the inflammatory pathway, including the nucleotide-binding oligomeric structural domain (NOD)-like receptor (NLR) family, which is an important component of the innate immune response (62). Recent studies have shown that the NLR family is involved in the inflammatory response during IVDD, the NLRP3 inflammasome is widely activated during IVDD, and the activated NLRP3 inflammasome is mainly involved in the inflammatory response, pyroptosis, ECM degradation and apoptosis in IVDD cells (63). A study reported that the expression of NLRP3, cysteinyl asparaginase-1 and IL-1 was significantly upregulated in patients with low back pain (64). Therefore, aberrant activation of the NLRP3 inflammasome can lead to an inflammatory cascade in IVDD, and the development of NLRP3 inflammasome inhibitors could be a new option for the treatment of IVDD.

#### 3.2.3 The complement system

The complement system is involved in innate immunity and can regulate the inflammatory cascade and clearance of noxious stimuli through three main pathways (classical, alternative or lectin) or a series of complement factors activated by the direct cleavage of C5 convertase to form the terminal complement complex (TCC) (65). The TCC was detected in human degenerated IVD tissues and was positively correlated with the grade of IVD degeneration (66). Furthermore, in a study of human degenerated CEP tissues, the lysosomal protease tissue proteinase D (CTSD), which is secreted by CEP, induced C5 cleavage, and C5a produced by this cleavage participated in the recruitment of immune cells (e.g., monocytes) that produced inflammatory cytokines that led to TCC deposition, activating the TCC cascade, and ultimately upregulating the expression of the catabolic enzymes MMP1 and ADAMTS4 in IVD cells, leading to IVDD (67). Therefore, the IVD inflammatory IVD microenvironment can be ameliorated by modulating the components of the complement system to alleviate IVDD. CTSD may be a potential candidate, and its inhibition may prevent ECM degradation and attenuate the complement activation-associated inflammatory response.

### 3.3 Adaptive immune response

#### 3.3.1 T cells

In IVDD, the adaptive immune response consists of cellular immunity involving T cells and humoral immunity involving B cells, and these effects are mediated by cells and antibodies, respectively (68). T cells have a TCR that receptor recognizes antigens when the APC and MHC molecules bind to each other. In contrast, CD8 T and CD4 T cells become CTLs and Th cells after recognizing MHC I and MHC II, respectively (69) (Figure 3).

Th cells play a crucial role in IVDD. In IVDD, T-cell-mediated cellular immune responses are activated by neovascular infiltration near the damaged IVD, while T cells migrate to the vicinity of the damaged IVD, recognize antigens and are activated and differentiate into Th1, Th2, and Th17 cells (70). Th1 cells secrete IL-2, IFN-γ, and other cytokines (71). Among them, IFN-γ is a potent proinflammatory cytokine (PIC) with multiple functions, including the induction of chemokine secretion and promotion of macrophage activation and phagocytosis, which in turn amplify adaptive immune responses (72, 73). In contrast, Th2 cells secrete anti-inflammatory cytokines (AICs), such as IL-4 and IL-10 (74). During the adaptive immune response, IL-4 contributes to antibody secretion by plasma cells and inhibits PIC secretion and macrophage activation (75, 76). Thus, Th1 and Th2 cells have opposite roles in regulating inflammatory responses. Interestingly, Th1 cells are mainly involved in cellular immunity, and Th2 cells are mainly involved in humoral immunity. In the normal IVD, cytokine production by Th2 cells predominantly suppresses cellular immune and inflammatory responses, and once the IVD is impaired, these factors interact with innate immune cells to promote Th1 cell production of proinflammatory factors and suppress Th2 cell responses, leading to an immune inflammatory response in IVDD (77).

**Figure 4:**
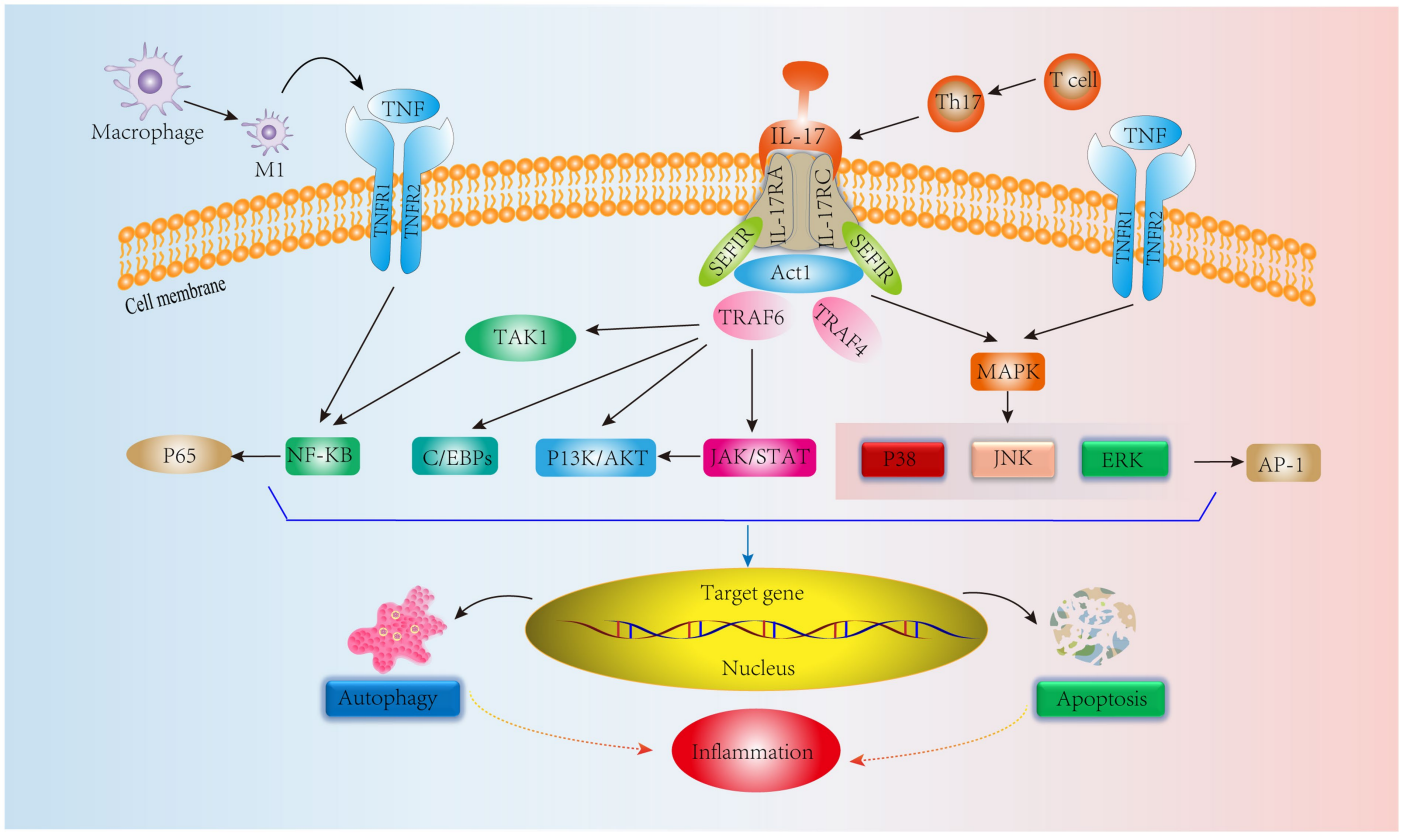
IL-17 cytokine production by Th17 cells, an adaptive immune response immune cell in IVD tissues, mediates relevant signaling pathways. In addition, TNF synergizes with IL-17 to stimulate NF-KB and MAPK signaling pathways leading to inflammation.

In addition, Th17 cells are equally important in adaptive immunity. Th17 cells produce IL-17, which amplifies the immune response to extrinsic environmental factors (78). Initiation of the IL-17, IL-17RA, and IL-17RC heterodimeric complex contributes to the immune inflammatory response (79). IL-17 signaling activates an NF-KB agonist (Act1), which consists of the Sef/IL-17R (SEFIR) structural domain and TNF receptor-associated factor 6 (TRAF6) binding motif (80). Yao et al. (81) showed that IL-17 could activate the NF-kB pathway, upregulate MMP-13 levels and decrease COL2 and ACAN expression, ultimately leading to ECM degradation and resulting in IVDD. Furthermore, in another human NPC experiment, IL-17A was found to upregulate COX2 levels and promote PGE2 secretion by activating the Ap1/c-Fos and JNK/c-Jun signaling pathways; the MAPK pathway includes P38, JNK, and ERK, and AP-1 is a downstream protein of the MAPK pathway (82). In addition, a study on IVD showed that IL-17 upregulated NPC vascular endothelial growth factor expression through the JAK/STAT pathway, thereby promoting IVD angiogenesis and exacerbating IVDD. Interestingly, JAK/STAT also induced PI3K/AKT activation to promote apoptosis and autophagy, which ultimately led to inflammation (83, 84). These results suggest that IL-17 is involved in IVDD through the NF-KB, MAPK, JAK/STAT, and PI3K/AKT signaling pathways. However, IL-17 alone has a weak effect on the inflammatory response, and it can act synergistically with other cytokines, such as TNF-α, to exacerbate the inflammatory response (85). Moreover, TNF-α can polarize macrophages to the M1 phenotype to exert proinflammatory effects (86). In addition to IL-17R, TNFR1/2 is involved in the IL-17 signaling response via the NF-KB and MAPK signaling pathways to activate the downstream proteins P65 and AP-1 to exert immunoinflammatory effects (87) (Figure 4). It is evident that TNF-α interferes with IL-17 signaling, affecting IVDD progression. Therefore, all T-cell-mediated adaptive immune responses directly or indirectly affect the IVD microenvironment, leading to IVDD. Based on these studies, we can see that delaying IVDD by inhibiting IL-17 cytokines or blocking its downstream pathway is not a new strategy.

#### 3.3.2 B cells

**Figure 5:**
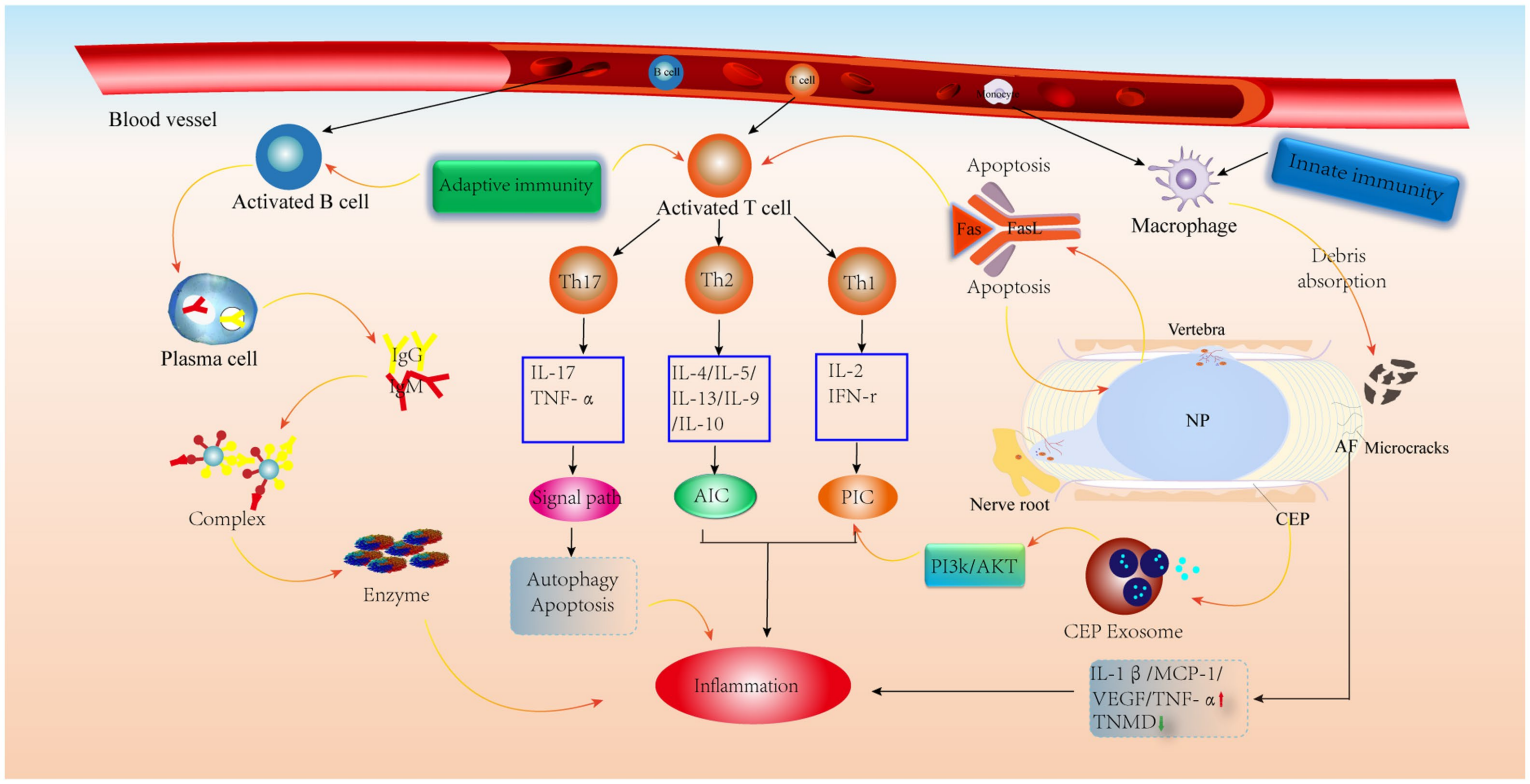
Schematic diagram of the immune anti-cascade response during IVD herniation. When the herniated NP tissue or AF, CEP develops microcracks and is recognized by the immune system, immune cells in the circulation are activated and released, along with AIC and PIC, which activate various signaling pathways leading to the catabolism of IVD cells. PIC, pro-inflammatory cytokine; AIC, anti-inflammatory cytokine; MCP-1, monocyte chemoattractant protein-1; TNMD, tenomodulin; VEGF, vascular endothelial growth factor.

Unlike T cells, B cells are activated by the BCR, recognize antigens, participate in B-cell differentiation and secrete antibodies to mediate humoral immunity (69). According to previous studies, some antibodies, such as IgG, have been found in IVD. Capossela et al. (88) identified type I, type II and V collagen and aggregated glycan-specific IgG in human degenerative IVD, and this study confirmed the correlation between IVDD and the immune response. Similarly, previous studies have confirmed the absence of antigen–antibody complexes in healthy IVDs and their presence in prominent IVD tissues (89). In addition, when the IVD is impaired, the NP is considered a hidden antigen that activates the immune system and contributes to the deposition of autoreactive immunoglobulin and complement attack complexes, thereby promoting chronic inflammation and ultimately leading to back pain (90). Moreover, serum levels of IgG and IgM are positively correlated with the degree of back pain in patients with degenerative IVD (91). IgM plays a role in adaptive immunity as the first defense in response to danger signals and as the first antibody to appear. Moreover, during conformational changes, IgM can activate the complement system to promote immune complex deposition, which mediates inflammation (92). In contrast, the IgG-mediated inflammatory response allows antigen–antibody immune complexes to enter the damaged IVD and phagocytose diseased tissue and normal cells, which then release lysosomal proteases to degrade the ECM and alter the biomechanical profile of the IVD, leading to IVDD (88, 90) (Figure 5).

#### 3.3.3 Autoimmune response

Because the IVD is avascular, it is considered an immune privileged site (93). The high concentration of proteoglycans in the CEP and its high physical stress microenvironment inhibit the inwards growth of blood vessels (94), which prevents immune cell recruitment. It has also been shown that CEP-derived exosomes activate the PI3K/AKT pathway to maintain CEP cell survival. In addition, a normal AF can inhibit angiogenesis by upregulating TNMD levels (95) and downregulating VEGF, TNF-α, IL-1β and MCP-1 levels (52). It is worth noting that the NP is always covered by the surrounding AF and CEP, preventing contact between the NP and the external environment (93). Furthermore, the expression of molecules with immunosuppressive effects on NP tissue keeps the NP in an immune-tolerant state, similar to the testis (96) and the eye (97). More importantly, FasL has been shown to promote apoptosis in vascular endothelial cells and contributes to immune tolerance in the NP (98–100). Therefore, these factors contribute to IVD immune exemption. However, when there are tiny cracks in the CEP or AF, these protective effects are weakened when the NP is exposed to the external environment, prompting vascular infiltration and thus triggering an autoimmune response (Figure 5).

## 4 IVDD immunomodulatory therapies

In recent years, regenerative medicine has emerged as an alternative approach to tissue and organ repair, and tissue engineering has emerged as a cross-disciplinary field with great potential for growth. Studies have shown that the combination of immunomodulation and tissue engineering lays the foundation for new therapies that contribute to tissue and organ recovery and regeneration (101). The immune system protects tissues and organs, guiding tissue repair, inducing tolerance and regulating autoimmunity (102). In addition, immunomodulation is essential for creating an environment for tissue regeneration, which allows for better functioning stem cells and bioactive molecules or substances that remain in the surrounding tissues to promote tissue repair and regeneration (103). The mutual integration of tissue engineering and immunomodulation in the context of therapeutic strategies combined with immunology has the potential to provide new strategies for tissue repair. Notably, surgery can lead to the loss of spine-related function, internal fixation breakage, and other complications. Therefore, the development of immunomodulatory therapies, such as mesenchymal stem cells, small molecules, growth factors, scaffolds, and gene therapy, is critical.

### 4.1 Mesenchymal stem cells

Mesenchymal stem cells (MSCs) are multifunctional and self-renewing cells in tissues such as bone marrow and adipose tissue (104, 105). In recent years, MSCs from different sources have been widely used to treat IVDD. Because MSCs do not express MHC-II, they are characterized as having low immunogenicity (106). Thus, MSCs are considered safe and effective and show great promise in IVD regeneration. MSCs have anti-inflammatory and immunomodulatory functions, and they secrete bioactive molecules to repair damaged tissues and modulate cellular immunity when they are in a suitable host. In the inflammatory environment of damaged IVDs, MSCs can recruit macrophages to the site of inflammation to promote IVD repair and immunomodulation. MSCs sensitize inflammatory factors and exert anti-inflammatory effects by switching macrophages from the M1 (proinflammatory) to the M2 (anti-inflammatory) phenotype through immunomodulators (e.g., prostaglandin E2) (107). On the other hand, MSCs can inhibit the proliferation and activation of T and B cells of any kind and tissue origin, which greatly attenuates the associated adaptive immune response (104). Furthermore, evidence suggests that bone marrow mesenchymal stem cells (BMSCs) can differentiate into NP-like cells and are resistant to catabolism (108). Similarly, in one study in which BMSCs were cocultured with NPCs in IVDD, NPC viability was increased, and apoptosis was significantly inhibited (109). Although significant breakthroughs have been made in preclinical aspects of MSC transplantation, it still has some limitations. First, the IVD is an avascular tissue, and transplanted MSCs are unable to obtain sufficient nutrients from the tissues surrounding the damaged IVD and die in large numbers; these dead cells undergo an inflammatory response and secrete many inflammatory cytokines, which affects the IVD microenvironment, resulting in an acidic environment and low glucose levels, which is often detrimental to cell survival (110). Second, leakage may occur during MSC injection, which can further exacerbate IVDD (111). Finally, the potential moral and ethical issues posed by MSC treatment, the associated immune rejection, and how to obtain sufficient quantities of MSCs are also pressing issues that need to be addressed (112).

**Figure 6:**
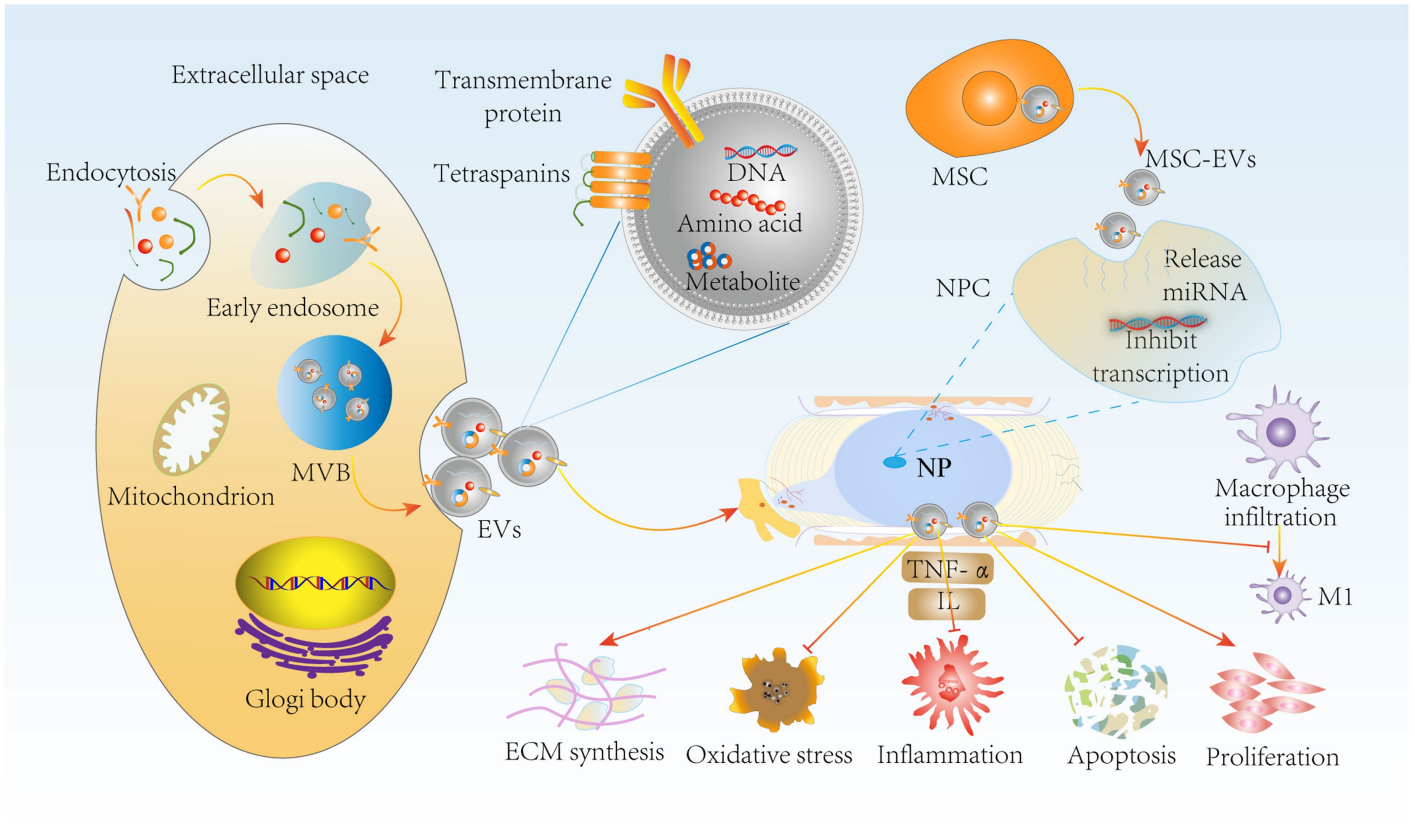
MSC-EVs delay IVDD through immunomodulatory effects. miRNA release by MSC-EVs inhibits NPC transfection through paracrine delivery on the one hand, suppressing NPC apoptosis, oxidative stress and inflammation. It also inhibited macrophage polarization to M1 type. On the other hand, MSC-EVs promoted NPC proliferation and ECM synthesis to maintain ECM homeostasis, further delaying IVDD progression. MSC-EVs, mesenchymal stem cell-derived extracellular vesicles; NPC, nucleus pulposus cells; MVB, multivesicular bodies.

Notably, extracellular vesicles produced by MSCs by paracrine means, such as MSC-EVs, can greatly reduce this side effect (113). MSC-EVs retain the functions of MSCs and escape *in vivo* immune rejection, providing some basis for tissue recovery. It has been reported that in a rabbit IVDD model, bone marrow mesenchymal stem cell (BMSC)-derived extracellular vesicles (BMSC-EVs) delayed IVDD by inhibiting the activation of NLRP3 inflammatory vesicles, which are involved in the innate immune response, and thus attenuated NPC apoptosis (114). Moreover, it has been shown that BMSC-EVs upregulate anabolic genes such as Col II and SOX and downregulate the catabolic gene MMP-3 to promote NPC proliferation and maintain ECM homeostasis (115). Furthermore, the secretion of inflammatory factors such as TNF-α, NF-κB P65, and IL is increased in IVDD, and adipose-derived mesenchymal stem cell (ADMSC)-derived extracellular vesicles (ADMSC-EVs) reduce the level of inflammation (116). More importantly, MSC-EVs inhibited the expression of the M1 macrophage phenotypic marker iNOS (117). Thus, the combination of immunomodulatory properties and the suppression of inflammatory responses by MSCs inhibits NPC apoptosis and oxidative stress, improves the IVD microenvironment and maintains ECM homeostasis (Figure 6). In conclusion, MSC-EVs, which are a novel therapeutic modality, have become a hot topic in the field of IVD regeneration due to their low immunogenicity, low tumorigenicity, high reparative properties, and nanoscale size for easy storage (118). In the future, we can use MSCs-EVs combined with drugs or carriers to obtain the desired results, such as injectable hydrogels for targeted delivery of drugs, which have resulted in IVD tissue repair and regeneration. However, the complexity of MSC-EV subpopulations makes it difficult to develop standardized dosages, and individual response variations are high (119). In addition, the complexity of IVDD pathology makes it challenging to select suitable donors of MSCs to generate appropriate amounts of extracellular vesicles.

### 4.2 Growth factors

Growth factors are multifunctional signaling molecules that coordinate the multistage process of tissue healing and are essential for tissue regeneration by directing cell behavior and promoting tissue repair and recovery (120). Studies have shown that the injection of growth factors into degenerated IVDs can modulate IVD homeostasis by promoting IVD cell proliferation, regulating ECM synthesis, inhibiting inflammatory responses, and downregulating mechanisms to degrade metalloproteinases (121, 122). These growth factors include BMPs and TGF-β. Among them, BMP-2 is a growth factor that efficiently induces bone tissue regeneration. Wei et al. (123) showed that BMP-2 significantly increased macrophage recruitment and migration and upregulated monocyte chemoattractant protein-1 (MPC-1) mRNA expression. MPC-1, in turn, is one of the key cytokines that regulate monocyte/macrophage migration and infiltration. Furthermore, in a rat study, BMP2 reduced ECM degradation, NPC apoptosis and the inflammatory response through the PI3K/Akt pathway (124). Therefore, controlling the activation of the immune response by regulating BMP-2 production, reducing monocyte/macrophage infiltration and chemokine production, and thus improving the IVD microenvironment may contribute to the recovery and regeneration of IVD tissue. Similarly, TGF-β plays an indispensable role in the regulation of the immune response; in particular, it promotes the survival of activated T cells (125). TGF-β can inhibit T-cell activation by blocking FasL-mediated apoptosis, and the Fas/FasL pathway is closely associated with the activation of autoimmune responses in NP tissues (126). Growth factors modulate the activation of T-cell adaptive immune responses through immunomodulation and reduce the immune-inflammatory cascade, effectively improving the IVD microenvironment and thus alleviating IVDD; however, studies have shown that the immunomodulatory and therapeutic effect of a single growth factor is often unsustainable to modulate IVDD (127). In the future, two or more growth factors can be used to compensate and regulate each other. In addition, the short half-life of growth factors makes it difficult to maintain effective concentrations for long periods of time, requiring repeated injections and excessive release, which inevitably increases the risk of complications and infections associated with the injections, making it difficult to use as an effective regenerative therapy (128). Therefore, the most important thing is to achieve controlled and slow release. In addition, growth factors are peptides that target cells (112). The low number of IVD cells in the advanced stages of IVDD makes growth factor injection difficult to implement clinically. Therefore, growth factor immunomodulatory therapy to treat IVDD is likely to be a promising therapeutic strategy in the regeneration of early regressed IVDs.

### 4.3 Small molecules

Small molecules are compounds that regulate specific biological processes and include synthetic or natural products (129), such as resveratrol, berberine, dexmedetomidine and naringenin. Resveratrol induces Bcl-2 expression and downregulates cystathionine 3 levels. In a rat CEP cell study, resveratrol significantly inhibited CEP cell apoptosis by inhibiting the HMGB1 pathway while reducing the immune cascade inflammatory response, which alleviated IVDD to some extent (130). It has also been shown that resveratrol can downregulate the NF-KB and P38 MAPK signaling pathways, modulating the immune inflammatory response and preventing disease progression. Berberine is another small molecule that significantly inhibited the activation of NF-KB in a human NPC assay, effectively exerting an antiapoptotic effect and blocking the immune inflammatory cascade activated by immune cell-cytokine interactions (131). In addition, pyroptosis can promote inflammation to a greater extent than apoptosis. This process is associated with activation of the NLRP3 inflammasome and innate immunity (132). Interestingly, dexmedetomidine is a small molecule sedative that inactivates the NLRP3 inflammasome by inhibiting NF-KB and JNK signaling (133), thereby reducing immune cell recruitment and cytokine release, improving the IVD inflammatory microenvironment and subsequently alleviating scorching during IVDD. There are also small molecules, such as naringenin, that have anabolic and anti-catabolic effects on IVD cells, which may have an impact on NPC regeneration by upregulating the expression of anabolic genes and decreasing the expression of catabolic genes, thus maintaining the ecological niche of the ECM and providing a beneficial microenvironment for the regeneration of IVD tissues (134). Therefore, small molecule drugs can modulate the IVD immune microenvironment to alleviate IVDD by inhibiting the immune inflammatory response, apoptosis, pyroptosis, and catabolism through different signaling pathways. It is evident that small molecule drugs show promising therapeutic potential in IVDD and the regeneration of IVD tissues.

Although small molecule drugs have shown good efficacy in the treatment of IVDD, the reports have been limited to *in vitro* studies. To date, these small molecule drugs have not been used in clinical trials. In addition, there is a lack of representative animal models for clinical translation, and the use of small experimental mammalian models, mainly rats and rabbits, to study IVD regeneration has certain drawbacks (135). For example, the persistence of the mechanical loading of IVDs varies, and there are significant differences in the degree of nutrient diffusion due to the lack of longitudinal compression in human IVDs, as well as the small size of IVDs (136). It is also important to focus on individualized treatment, as well as the pharmacology of the drug, when using small molecule drugs. These small molecule drugs may be indicated only in the early stages of IVDD. Therefore, it is important to assess the type and stage of IVDD before starting drug therapy to determine the timely response of the body to small molecule drugs (137). Finally, the avascular nature of IVDs may = increase the duration of sustained treatment with small molecule drugs. It is worth noting that small molecule drugs may be applied systemically and may have more side effects or difficulty in achieving effective concentrations in some specific cases. In the future, we can use microspheres or hydrogels for *in situ* controlled release of small molecule drugs for local delivery as a new strategy to overcome the deficiencies of systemic delivery, to achieve effective therapeutic concentrations and a long duration of treatment.

### 4.4 Scaffolds

Tissue engineering is primarily achieved by properly combining cells with appropriate scaffolding materials and bioactive signaling molecules to improve or restore the function of damaged tissues (138). The use of scaffold materials offers great promise for tissue healing and regeneration. Remarkably, scaffolding materials can mimic the 3D spatial structure of natural tissues by using cells, drugs, molecules and growth factors to modify scaffolds and ultimately modulate cell behavior and immune responses (103). Interestingly, these scaffolds can also modulate innate and adaptive immune responses by promoting macrophage polarization to an M2 phenotype and inhibiting dendritic cell maturation (139). In addition, tissue engineering treatments have stringent requirements for scaffold materials, including low immunogenicity and good biocompatibility with the host. However, a single biomaterial cannot meet the criteria for IVD regeneration, and at least two or more biomaterials are typically needed. For example, composite hydrogels made of nanofibers and chitosan can be used for IVD reconstruction, repair and regeneration; the former mainly provides mechanical properties similar to those of collagen fibers in IVDs, while the latter mainly provides low immunogenicity, cytocompatibility, nontoxicity and easy degradation (140). However, a single biomaterial cannot meet the requirements of IVD regeneration, and at least two or more biomaterials are often needed. For example, cellulose nanofiber-filled chitosan composite hydrogels can be used for IVD reconstruction, repair and regeneration; the former mainly provides mechanical properties similar to those of collagen fibers in IVDs, and the latter mainly provides low immunogenicity, cytocompatibility, nontoxicity and easy degradation (141). In addition, chitosan and alginate biomaterials can be used to treat IVDD by modulating the immune inflammatory microenvironment and by carrying immunomodulatory agents. Recently, an injectable chitosan hydrogel-loaded celecoxib delivery system with a porous structure that provides three-dimensional space to accommodate and protect the prolonged controlled release of celecoxib was designed and used in animal experiments to reduce the local inflammatory response and proinflammatory cytokine production, and chitosan can polarize macrophages around damaged IVDs to the M2 phenotype without causing T-cell proliferation and activation (142, 143), thereby reducing the activation of the adaptive immune response and immune cell-mediated inflammatory cascade response to effectively delay IVD degeneration. It has also been shown that the synergistic effects of silk gliadin and graphene oxide in injectable alginate hydrogels significantly promoted M2 macrophage infiltration and the proliferation of MSCs, and an increased regenerative immune response was observed in response to the incorporation of bioactive molecules (144). In addition, *in vivo* transplantation with ultrapure alginate (UPAL) gel combined with BMSCs after partial IVD resection in a rabbit model promoted the differentiation of BMSCs into NPCs, as well as growth factor and ECM production for IVD regeneration (145). Since hydrogels cannot effectively control drug release, controlled release can be achieved by combining them with microsphere carriers. Recently, an OPF/SMA hydrogel scaffold loaded with dual-drug/slow-release PLGA microspheres containing IL-4 (IL-4-PLGA) and kartogenin (KGN-PLGA), which is a novel injectable composite hydrogel scaffold, was designed. IL-4-PLGA induced macrophage polarization to an M2 phenotype, whereas KGN-PLGA exerted sustained anti-inflammatory effects. More importantly, the experiments confirmed the hypothesis that the IVD immune microenvironment could be improved by sequential drug release for the purpose of repairing NP tissues. The experimental results also showed that the combination of stent material and immunomodulation was very effective in treating IVDD (146). Therefore, we will explore the molecular mechanisms of macrophage polarization to study IVDD in the future. Although tissue engineering scaffolding materials have the potential to modulate the IVD inflammatory microenvironment for the treatment of IVDD, we must note that most IVDD studies simulate the condition *in vitro*, and there is a lack of *in vivo* studies of large animals similar to humans (147). Moreover, the selection of suitable donor cells should enable survival and function in unfavorable inflammatory microenvironments. In conclusion, combined tissue engineering scaffolds and immunomodulation therapy can greatly improve the efficiency and efficacy of regenerative immunotherapy for IVD. To some extent, reducing the infiltration and activation of immune cells, decreasing the inflammatory cascade response, improving degenerative IVDs and remodeling the microenvironment of IVD cells could be a suitable strategy for the clinical treatment of IVDD.

### 4.5 Gene therapy

Gene therapy involves the insertion of exogenous genes into appropriate recipient cells of IVDD patients via vectors, which provide sustained therapeutic effects once gene transfer is successful (148). Essential vectors contribute to translocation into the nucleus and the expression of transgenes, including AAV and RNAi (147). Among them, AAV has attracted much interest from researchers. In a recent study of a rat IVD model, injection of a highly expressed TGF-β1 AAV vector into the rat IVD resulted in TGF-β1-mediated promotion of T-cell activation, and ACAN and Col-2 levels were upregulated in NP tissues, while Smad3 levels were downregulated, thus promoting rat IVD cell proliferation and alleviating NPC senescence, which are essential for restoring the IVD microenvironment (149). Recent studies have shown that the expression of hub genes (ID1, PTPRK and RAP2C) is positively correlated with TNF secretion. As the degree of IVD degeneration increases, the infiltration of immune cells increases, as does the expression of hub genes. Damaged IVD sites recruit immune cells, such as macrophages, which interact with NPCs through cytokines such as TGF-β and IL-10 and promote abnormal expression of the hub genes ID1, PTPRK and RAP2C in NP cells, leading to pathological changes in the IVD (150). Therefore, an immunomodulatory approach can reduce the production of inflammatory cytokines and reduce immune cell recruitment to improve the IVD microenvironment, which in turn downregulates the expression of these pivotal genes and mitigates IVDD. Gene therapy affects IVDD by indirectly participating in the immune response and altering the survival and activity of immune cells. Although there have been breakthroughs in modulating IVD activity through the delivery of therapeutic genes, there are some obvious problems. First, most of the research is still in the laboratory setting. Second, gene therapy involves viral vectors, which may carry the risk of immune responses and associated complications such as viral mutations. Therefore, well-designed gene therapy experiments have great potential in the treatment of IVDD (Table 1).

**Table 1:** IVDD-related immunomodulatory therapy.

Immunomodulatory therapy	Component	Pathway	Function	References
Mesenchymal stem cell	BMSCs ADMSCs-EVs	Immunomodulator PGE2; BMSCs-EVs;ADMSCs-EVs	Promotes macrophage M1 polarization to M2 phenotype; inhibits proliferation and activation of T and B cells; inhibits NPC apoptosis and oxidative stress and promotes ECM synthesis; inhibits inflammation and modulates the immune microenvironment	Melief et al. (107), Zbinden et al. (104), Xia et al. (114), Li et al. (115), Wei et al. (117)
Growth factors	BMP-2; TGF-β	MPC-1; PI3K/Akt signaling pathway and Fas/FasL pathway	Reduces production of immune cells and chemokines; modulates T-cell adaptive immune response activation and reduces the immune-inflammatory cascade	Wei et al. (123), Tan et al. (124), Cerwenka et al. (126)
Small molecule	Resveratrol, berberine, dexmedetomidine and naringenin	HMGB1; NF-KB; P38 MAPK; NLRP3 inflammatory inflammasome and JNK signaling pathway	Suppression of immune cascade inflammatory response, apoptosis, cellular pyroptosis and catabolism	Hu et al. (130), Lu et al. (131), He et al. (132), Zhou et al. (133), Zhang et al. (134)
Scaffolds	Chitosan; Alginate; OPF/SMA; PLGA	Injectable chitosan hydrogel-loaded celecoxib delivery system; UPAL gel combined with BMSCs for *in vivo* transplantation; dual-drug/extended-release PLGA microspheres	Enhances IVD cell proliferation and tissue regeneration; modulates the immune-inflammatory microenvironment and carries immunomodulators; reduces local inflammatory responses and inflammatory cytokine production; inhibits T-cell proliferation and activation and reduces immune cell infiltration and activation	Du et al. (142), Kim et al. (143), Ukeba et al. (145), Cheng et al. (146)
Gene therapy	AAV and RNAi	Low-expressing AP-2α and high-expressing TGF-β1AV vectors injected into rat IVD; hub gene	Downregulates the expression of MMP-2, MMP-9 and Smad3 and upregulates the expression of ACAN and Col-2; alters the survival and activity status of immune cells	Xia et al. (147), Li et al. (149), Wang et al. (150)

## 5 Summary and outlook

In this article, we explored the levels of various immune cells, signaling pathways and cytokines in IVDD from an immunomodulatory perspective and analyzed the interactions between immunomodulatory therapies for IVDD. Immunomodulation works through multiple mechanisms to counteract inflammatory responses and maintain IVD tissue homeostasis to avoid injury. These mechanisms work in concert in a fully integrated immune system to respond to specific IVD injuries. Abnormal regulation of various effector mechanisms can lead to alterations in the IVD microenvironment. Therefore, understanding the fundamental relationships between different immune effector pathways is essential for the treatment of IVDD. The IVD, which is an immune privileged organ, develops progressive IVDD when subjected to aberrant mechanical loads, oxidative stress, and trauma. On the one hand, AFCs or NPCs are targets of immune system attack in response to contact with the external environment, triggering an immune response that results in the formation of an antigen–antibody-complex reaction; On the other hand, extruded or herniated NPs may also compress the nerve root. These factors recruit immune cells, such as monocytes/macrophages and T cells, to remove necrotic tissues and cellular debris while releasing large amounts of cytokines and chemokines that activate various downstream signaling pathways, leading to a severe degenerative inflammatory cascade. Therefore, immunomodulatory therapies can improve the IVD microenvironment to repair IVDD and are a viable treatment modality. This will ultimately contribute to the discovery of suitable drugs to provide more effective treatments for most IVDD patients.

A growing body of evidence demonstrates the potential of immunomodulatory therapies in the treatment of IVDD. These therapies aim to halt the progression of IVDD and restore the integrity of the IVD microenvironment. However, current immunomodulatory therapies are mainly indicated in the early stages of IVD regression and have limited therapeutic efficacy in the treatment of advanced IVDD. Therefore, early intervention to prevent IVDD progression is crucial. To bridge the gap between experimental animal models and clinical trials, we need to use animal models that are more similar to the clinic and better mimic the complexity of human IVD and the pathophysiology of IVDD. With the development and application of immunomodulation to treat IVDD, we need to delve deeper into the mechanisms of IVDD degeneration and integrate pathophysiology with immunomodulatory therapy to promote the clinical application of immunomodulatory therapy for IVDD and improve patient quality of life.

## Author contributions

YG: Writing – original draft. XC: Writing – review, Methodology & Resources. GZ: Funding acquisition, Supervision, Writing – review & editing, Validation. ML: Writing – review & editing, Supervision, Visualization. XZ: Conceptualization, Funding acquisition, Supervision, Writing – review & editing. HZ: Resources, Writing – review & editing.
